# Endocrine-related adverse events in a large series of cancer patients treated with anti-PD1 therapy

**DOI:** 10.1007/s12020-021-02750-w

**Published:** 2021-05-25

**Authors:** Rossella Rubino, Andrea Marini, Giandomenico Roviello, Elena Margherita Presotto, Isacco Desideri, Isabella Ciardetti, Marco Brugia, Nicola Pimpinelli, Lorenzo Antonuzzo, Enrico Mini, Lorenzo Livi, Mario Maggi, Alessandro Peri

**Affiliations:** 1 Endocrine Unit, Department of Experimental and Clinical Biomedical Sciences “Mario Serio”, University of Florence, AOU Careggi, Florence, Italy; 2 Unit of Translational Oncology, AOU Careggi, Department of Health Sciences, Section of Clinical Pharmacology and Oncology, University of Florence, AOU Careggi, Florence, Italy; 3 Radiation Oncology Unit, Oncology Department, AOU Careggi, Department of Experimental Clinical and Biomedical Sciences “Mario Serio”, University of Florence, Florence, Italy; 4 Melanoma & Skin Cancer Unit, Tuscany Central District; Department of Health Sciences, Dermatology Unit, University of Florence, Florence, Italy; 5 Medical Oncology Unit, AOU Careggi, Florence, Italy; 6 Department of Experimental and Clinical Medicine, University of Florence, Florence, Italy

**Keywords:** Endocrine toxicity, Cancer, Immunotherapy, Anti PD-1

## Abstract

**Purpose:**

Immune checkpoint inhibitors have opened a new scenario in the treatment of cancer. These agents can elicit adverse events, which may affect different systems and organs, including the endocrine system. The aims of this study were to evaluate the impact of the anti-PD-1 molecules nivolumab and pembrolizumab on endocrine toxicity and on patient outcome.

**Methods:**

A retrospective and multicentre study was designed, which involved a total of 251 patients affected by different tumors (mostly non-small cell lung cancer, 68.92% and melanoma, 24.30%) and treated with the PD-1 inhibitors nivolumab (61.35%) or pembrolizumab (38.65%) for up to 60 months. Clinical and biochemical data were recorded until July 31, 2020.

**Results:**

Endocrine toxicity occurred in 70 out of 251 patients (27.89%). It was mostly related to thyroid dysfunction and in 75% of cases occurred within 6 months from the beginning of therapy. A previous endocrine morbidity and female gender were predictors of endocrine toxicity. There was no association between endocrine dysfunction and patient outcome. However, when all toxicities (i.e., endocrine and non endocrine) were considered, a significant association with progression-free survival and overall survival was found.

**Conclusions:**

Thyroid alterations are frequently observed in cancer patients treated with anti PD-1 drugs, particularly in women and in the presence of a previous endocrinopathy. We suggest that regular thyroid assessment should be performed in these patients, especially in the first months of therapy. Finally, the onset of side effects, related to anti PD-1 agents, appears to be associated with a better outcome.

## Introduction

Immune checkpoint inhibitors (ICPIs) opened a new era in the treatment of advanced cancer. ICPIs are monoclonal antibodies that inhibit negative regulatory pathways of the immune response, ultimately stimulating T cells to attack cancer cells. The T lymphocytes-associated antigen 4 (CTLA-4), the programmed cell death protein-1 (PD-1) and its ligands (PD-L1/PD-L2) are the targets that have been utilized in recent years, in order to design new pharmacological strategies [1]. Historically, the CTLA-4 inhibitor ipilimumab was the first ICPI approved in the U.S. for the treatment of advanced-stage melanoma. PD-1 inhibitors (e.g., nivolumab, pembrolizumab) and PD-L1 inhibitors (e.g., atezolizumab, avelumab, durvalumab) followed and the spectrum of indications has widened to different tumors, including, for instance, non-small cell lung cancer (NSCLC), renal cell carcinoma, Hodgkin lymphoma, head and neck squamous cell cancer, hepatocellular carcinoma [2–9].

Because of their mechanism of action, ICPIs can be associated with immune-related adverse events (irAEs). The most commonly reported irAEs involve the respiratory and gastrointestinal apparatus, the skin, the endocrine glands (e.g., pituitary, thyroid, adrenal, endocrine pancreas). Hypophysitis can occur during immunotherapy and is more commonly observed in patients treated with ipilimumab [10, 11]. Thyropathies are more frequently associated with anti-PD-1 treatment or with a combination of anti-PD-1 and anti-CTLA4 [10, 11]. Insulin-deficient diabetes mellitus and primary adrenal insufficiency are rarely reported [10, 11]. With regard to thyroid dysfunction, which is overall the most frequent endocrine irAE, both hypothyroidism or thyrotoxicosis may occur. The latter can be transient and can be followed by subsequent hypothyroidism [12, 13].

The aim of this retrospective, multicentre study was to analyze: (i) the occurrence and the course of endocrine irAEs in a series of 251 patients with different cancers treated with the PD-1 inhibitors nivolumab or pembrolizumab, and (ii) the relationship between the onset of irAEs and outcome.

## Patients and methods

### Patients

Two hundred and fifty-one patients consecutively referred to reference Centres in the Florence area (e.g., Radiation Oncology Unit, Medical Oncology Unit and Unit of Translational Oncology at the AOU Careggi; Melanoma & Skin Cancer Unit, Tuscany Central District, IOT Hospital) and treated with nivolumab or pembrolizumab for advanced cancer disease were enrolled in this retrospective study. Consent for the data collection was collected from each patient. The study was approved by the Careggi Hospital Ethics Committee. Clinical data from October 1, 2017 to July 31, 2020 were recorded for patients with a follow-up of at least 2 months from the initiation of immunotherapy. The followup ranged from 2 to 60 months. Nivolumab was administered i.v. (usually 3 mg/kg) every 2 weeks, pembrolizumab was administered i.v. (usually 2 mg/kg) every 3 weeks. Table 1 shows the demographic characteristics of the patients. Biochemical data, including thyroid function, cortisol, glycemia, serum electrolytes at baseline and during ICPIs therapy were collected from clinical charts. All the hormone and biochemistry measurements were performed at the central lab of the Careggi Hospital. Normal plasma levels were within 0.36–3.74 mU/L for TSH, 9.8–18.8 pmol/L for fT4, 3.3–6.1 pmol/L for fT3, 138–635 nmol/L for cortisol, 65–110 mg/dL for glycemia. Blood samples had been drawn after an overnight fast before every treatment with anti-PD-1. All the blood parameters were measured according to a routinary screening of possible complications of ICPIs treatment. In patients who were receiving corticosteroids at some point during follow up (about 15–20%, yet only 3–4% of cases at high doses for the treatment of severe toxicities), blood serum cortisol was assessed once treatment had been discontinued.

**Table 1:** Demographic characteristics of the patients

	Males	Females	Total
Number	155	96	251
Age (years)	71.63 ± 1.57 (range 46–93)	68.93 ± 2.15 (range 44–95)	70.60 ± 1.28 (range 44–95)
Tumor			
NSCLC	109 (70.32%)	64 (66.66%)	173 (68.92%)
Melanoma	38 (24.52%)	23 (23.96%)	61 (24.30%)
Kidney	7 (4.52%)	3 (3.12%)	10 (3.98%)
Breast	0	4 (4.16%)	4 (1.59%)
Head-Neck	1 (0.64%)	1 (1.04%)	2 (0.80%)
Bladder	0	1 (1.04%)	1 (0.40%)
ICPI			
Nivolumab	94 (60.64%)	60 (62.50%)	154 (61.35%)
Pembrolizumab	61 (39.35%)	36 (37.50%)	97 (38.65%)

### Statistical analysis

Continuous variables with a normal distribution and categorical variables were expressed as mean ± 95% confidence interval. Differences between groups in continuous and categorical variables were assessed by Student *t* and chi-squared test, respectively. The simultaneous analysis of all possible predictors of endocrine toxicity was performed by binary logistic regression. The analysis aimed to assess the possible association between five putative predictors and the occurrence of endocrine alterations, with “no endocrine toxicity” or “endocrine toxicity” being the dependent variable.

The trend over time of thyroid toxicity was evaluated by a survival analysis using the log-rank test to evaluate the difference in events among the months of observation.

The correlation between the onset of toxicities and outcome was analyzed using chi-squared test. Progression free survival (PFS) and overall survival (OS) were represented using Kaplan–Meier analysis and compared using log-rank test.

## Results

### Endocrine Toxicity

Endocrine irAEs were registered in 70 out of 251 patients (27.89%). Of these 70 patients, 36 were males (51.43%) and 34 (48.57%) were females. The follow up ranged from 2 months to 60 months (median 18.48 ± 1.7, 95% CI) (Table 2). With regard to the type of tumor in patients with endocrine toxicity, 53 (75.71%) patients had NSCLC, 13 (18.57%) melanoma, 2 (2.85%) renal cancer, 1 (1.43%) breast cancer and 1 (1.43%) bladder cancer. Forty-two (60%) of these patients were treated with nivolumab and 28 (40%) with pembrolizumab. In the large majority of cases, endocrine toxicity was related to a thyropathy (*n* = 66, 94.28%). It is known that also tyrosine kinase inhibitors, which may precede immunotherapy, may be associated with thyroid dysfunction or may affect the onset of ICPI related toxicity [14, 15]. However, in our series, only 6 patients had been previously treated with tyrosine. Kinase inhibitors and thyroid alterations had not been reported. Besides thyroid dysfunction, in two patients (2.86%) the onset of type 1 diabetes mellitus was reported and insulin treatment was started. In two other cases (2.86%) secondary hypocortisolism or hypothyroidism were observed.

**Table 2:** Patients on follow-up at different times

Months	N. of patients
1	251
2	251
3	246
4	237
5	226
6	215
12	182
24	99
36	48
48	18
60	5

The analysis of the presence of possible risk factors for the development of endocrine irAEs showed that a pre-existing endocrinopathy was a strong predictor (Table 3). A gender difference was also observed, namely women had a higher risk to develop an endocrinopathy during treatment with nivolumab or pembrolizumab than men. Conversely, the type of cancer, the type of anti PD-1 and age were not predictors of endocrine toxicity in our series of patients. Moreover, a pre-existing endocrinopathy was confirmed as a significant predictive factor even when analyzed separately in males and females (not shown).

**Table 3:** Predictive factors for the onset of endocrine toxicity during treatment with nivolumab or pembrolizumab

Predictor	Toxicity				Chi2	*p*
	No		Yes			
*Gender*						
Male	119	76.8%	36	23.2%	4.381	0.036
Female	62	64.6%	34	35.4%		
*Type of tumor*						
Head-Neck	2	100.0%	0	0.0%	5.647	0.342
Breast	3	75.0%	1	25.0%		
Melanoma	48	78.7%	13	21.3%		
NSCLC	120	69.4%	53	30.6%		
Kidney	8	80.0%	2	20.0%		
Bladder	0	0.0%	1	100.0%		
*Age*						
Average age	70.52	n/a	70.8	n/a	0.176	0.860
*Nivolumab vs Pembrolizumab*						
Nivolum ab	112	72.7%	42	27.3%	0.075	0.784
Pembrolizumab	69	71.1%	28	28.9%		
*Previous endocrine disease*						
No	156	78.4%	43	21.6%	18.840	0.00001
Yes	25	48.1%	27	51.9%		

The above-mentioned predictors were further analyzed simultaneously in a unique statistical model using the binary logistic regression in order to assess a possible interaction between each predictor. The results showed that a pre-existing endocrinopathy remained a significant predictor, with a 53.9% probability of occurrence of endocrine toxicity during immunotherapy (Table 4).

**Table 4:** Simultaneous analysis of all possible predictors of endocrine toxicity by binary logistic regression analysis

		Statistical significance	Odd ratio	Odd Ratio C.I. (95%)		Probability	Probability C.I. (95%)	
				Lower	Upper		Lower	Upper
Gender	Male	0.074	1.717	0.95	3.103	0.233	0.215	0.250
	Female					0.365	0.334	0.395
Tumor	NSCLC	0.284	0.799	0.53	1.204	0.298	0.277	0.320
	Melanoma					0.247	0.215	0.279
	Kidney					0.276	0.189	0.363
	Breast					0.320	0.177	0.464
	Head-Neck					0.125	0.073	0.177
	Bladder					0.120	0.120	0.120
Previous endocrinopathy	No	**<0.001**	4.156	2.141	8.065	0.216	0.209	0.223
	Yes					**0.539**	0.518	0.560
Anti PD-1	Pembrolizumab	0.902	1.039	0.57	1.894	0.289	0.260	0.318
	Nivolumab					0.279	0.257	0.302
Age	≤50	0.683	0.958	0.782	1.175	0.289	0.207	0.372
	51–55					0.250	0.214	0.285
	56–60					0.278	0.217	0.340
	61–65					0.311	0.258	0.363
	66–70					0.270	0.227	0.312
	>70					0.286	0.260	0.311

A result is considered statistically significant if it allows to reject the null hypothesis. A significance level of 5% was considered, namely all the results with a *p* value < 0.05 have a 5% probability that the null hypothesis is correct. Conversely, the same results have a 95% probability that the expected result is correct

*C.I.* Confidence Interval

**Fig. 1:**
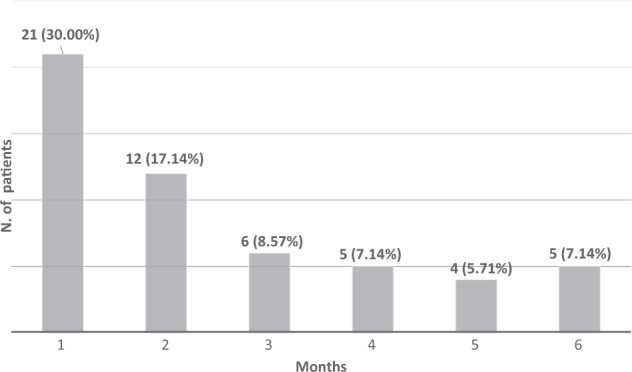
Time of onset of endocrine toxicity. The % refers to the total number of patients in which endocrine toxicity was found (*n* = 70)

In 33 patients (47.14%) endocrine toxicity was present within 2 months from the initiation of immunotherapy and in 53 (75.71%) within the first 6 months (Fig. 1). No difference in the time of appearance of toxicity was observed between patients with or without a pre-existing endocrinopathy (15.14 ± 5.92 *vs* 16.81 ± 4.55 weeks, respectively, *p* > 0.05).

### Thyropathy

Among the 66 patients who presented a thyropathy, 38 (73.68%) had no previous history of endocrinopathies, whereas 28 (26.32%) had already a previous endocrine disease (i.e., a prexisting thyroid alteration or diabetes mellitus). The most frequent thyroid alteration was hypothyroidism (*n* = 34, 51.52%), followed by thyrotoxicosis (*n* = 17, 25.76%) and transient thyrotoxicosis followed by hypothyroidism (*n* = 15, 22.72%). In more than half of patients (*n* = 36, 54.5%) thyroid alteration was mild and did not require specific pharmacological intervention, whereas in 30 cases (45.5%) thyroid specific treatment was started or dose adjustment was needed.

### irAEs and outcome

**Fig. 2:**
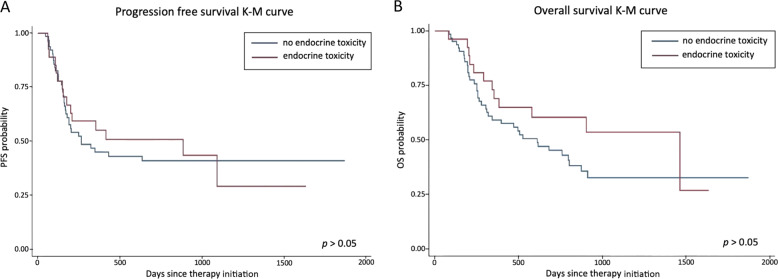
Association between endocrine toxicity and clinical outcomes. Kaplan–Meier plots of (**A**) progression-free survival (PFS) and (**B**) overall survival (OS) in patients who developed endocrine toxicity *vs* patients who did not

**Fig. 3:**
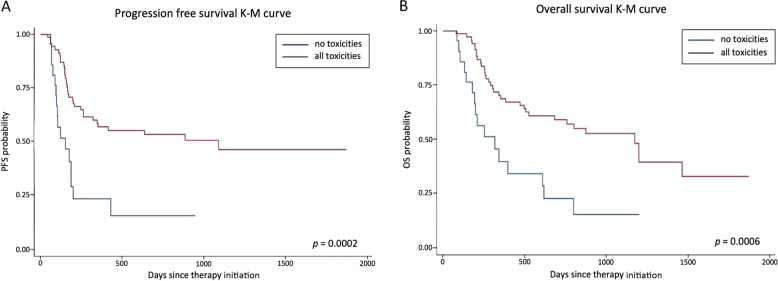
Association between all toxicities and clinical outcomes. Kaplan–Meier plots of (**A**) progression-free survival (PFS) and (**B**) overall survival (OS) in patients who developed toxicities *vs* patients who did not

In order to assess whether endocrine toxicity was associated with a different outcome in our series of cancer patients treated with nivolumab or pembrolizumab, Kaplan–Meier estimates were analyzed in a homogeneous subgroup of patients with NSCLC (*n* = 92), who had started immunotherapy by December 31, 2018 and had received at least four treatment cycles. Neither PFS, nor OS significantly differed between patients who presented (*n* = 27) or not (*n* = 65) endocrine toxicity (median: 887 *vs* 285 days, PFS, *p* > 0.05; 1465 *vs* 612 days, OS, *p* > 0.05, respectively) (Fig. 2). However, when all toxicities (i.e., including non-endocrine toxicities, Table 5) were considered, PFS and OS were significantly longer in patients who presented irAEs (*n* = 71), compared to those who did not (*n* = 21) (median: 1093 *vs* 154 days, PFS, *p* = 0.0002; 1200 *vs* 320 days, OS, *p* = 0.0006, respectively) (Fig. 3), even though in 18 cases immunotherapy had to be temporarily withheld (*n* = 9) or definitively interrupted (*n* = 9).

**Table 5:** Non-endocrine-related toxicities in the subgroup of patients with NSCLC (*n* = 92) treated with immunotherapy

Toxicity	Number of cases
Fatigue	31
Skin rash	18
Itchiness	16
Diarrhea	15
Hypertransaminasemia/hyperbilirubinemia	11
Pneumonia	10
Hyporexia/anorexia	10
Arthritis	8
Mucositis	7
Fever	6
Nausea	6
Myalgia	4
Xerostomia	3

## Discussion

In this multicentre study, we have addressed irAEs in a large series of cancer patients treated with anti-PD1 agents. We analyzed medical records from 251 patients (155 males and 96 females) affected by different tumors, mainly NSCLC (68.92%) and melanoma (24.3%). The median age was 70.6 years, with a range from 44 to 95 years. Patients received nivolumab (61.35%) or pembrolizumab (38.65%) and the follow-up ranged from 2 to 60 months.

Endocrine toxicity was observed in 27.89% of patients (*n* = 70). Endocrine irAEs are among the most frequent toxicities. An analysis of reports and case series published in the literature from January 2016 to April 2018 retrieved, among the 101 publications included, 139 cases of irAEs and 59 of these (42.4%) were identified as endocrine toxicities, including thyroid, adrenal, pancreas alterations [16]. Endocrine alterations may turn out to be complex toxicities and some of them (e.g., hypocortisolism, diabetic ketoacidosis) may be life-threatening, if not recognized and appropriately treated. Data from the FDA Adverse Events Reporting System database revealed 6260 records related to endocrine irAEs during ICPLs treatment. Life-threatening situations occurred in 7.57% of cases and death in 9.6% [17].

With regard to the type of tumor in patients presenting endocrine toxicity, the majority of cases were registered in patients with NSCLC (75.71%) or melanoma (18.57%), which were the most represented tumors in our database. If we consider the total number of patients with NSCLC or melanoma included in the study, we observed endocrine toxicity in 30% and 21% of these tumors, respectively. As per the specific treatment in patients with NSCLC or melanoma, 42 patients (60%) were treated with nivolumab and 28 (40%) with pembrolizumab, virtually the same distribution between the two treatments in the total group of 251 patients included in the study.

In almost all cases endocrine irAEs were related to thyroid toxicity (*n* = 66, 94.28%). In the remaining four cases, we observed two patients who developed diabetes mellitus, which required insulin treatment, and two patients who developed pituitary alterations (secondary hypothyroidism and secondary hypocortisolism). It is likely that hypophysitis caused pituitary dysfunctions in these two patients; however, no MRI or anti-pituitary Abs were available. Anyway, a TC scan of the brain, which included a detailed analysis of the pituitary gland and of the pituitary stalk, excluded the presence of potentially metastatic lesions. These data are in agreement with previously published original studies and reviews, which addressed thyroid toxicity as the most frequent endocrine complication of cancer immunotherapy, particularly with anti-PD1 molecules [10–13]. Interestingly, the thyroid gland appears the preferred target of drugs that modulate the immune response in diseases other than cancer. This is the case for instance of alemtuzumab, a humanized monoclonal Ab that targets CD52^+^ cells, in patients with multiple sclerosis [18]. It has been suggested that the preferential onset of thyropathies during treatment with immunomodulatory drugs may be due to the prevalence of anti-thyroid Ab in the general population (up to 18%) and to genetic factors that predispose to thyroid autoimmunity [19].

We then analyzed the presence of possible predictors of endocrine toxicity. We found, in agreement with a previous report, that a pre-existing endocrinopathy is a significant predictor of the development of endocrine irAEs [13]. We also observed a gender difference, with a significantly higher risk to present endocrine toxicity in women. Conversely, the type of cancer, the type of anti PD-1 and age were not predictors of endocrine alterations related to anti-PD1 therapy in our series of patients. When these parameters were analyzed simultaneously in a unique statistical model by binary logistic regression, a pre-existing endocrinopathy remained a significant predictor of the occurrence of endocrine toxicity during treatment with nivolumab or pembrolizumab.

An early onset of endocrine toxicity, i.e., within 2 months since the initiation of therapy, was reported in almost half of cases; in three out of four patients endocrine alterations were observed within 6 months. An early occurrence from the first dose of anti-PD1 and the onset of thyroid alterations has been reported in previous studies [20, 21]. Therefore, it is worth recommending that endocrine alterations, and mostly thyroid dysfunction, should be routinely investigated since the beginning of immunotherapy with anti-PD1 molecules.

As previously mentioned, thyroid alterations were by far the most frequent endocrine irAEs observed in our study. This finding is in agreement with clinical experience, which shows that thyroid disorders are the most common endocrine alterations during cancer treatment with anti PD-1 agents [10]. The most frequent thyroid alteration was represented by hypothyroidism (51.52%), followed by thyrotoxicosis or transient thyrotoxicosis with a subsequent shift into hypothyroidism. These three different patterns of thyroid disease presentation have been described in other studies [13, 15, 20–25]. In more than half of patients with a thyropathy (54.5%), a mild clinical presentation, which did not require specific treatment, was observed. Conversely, in 45.5% of cases thyroid specific therapy was required, either as a new therapy or as a dose adjustment of an ongoing treatment.

The etiophathogenesis of thyroid alterations associated with immune-check point inhibitors is still unclear. In particular, it has not been clarified yet whether anti-thyroid Ab play a role in the development of thyroid alterations. This is at least partially due to the fact that anti-thyroid Abs have been rarely determined in the published studies, so far. Admittedly, thyrotoxicosis in this setting is usually mild and transient and there is no recommendation to measure Abs [10]. In our series of patients, anti-thyroid Abs had been checked only in a very few cases and no specific comment is possible. Nevertheless, the presence of thyroid Ab does not appear a crucial factor for the development of thyroid dysfunction [25–28], thus suggesting that thyroid toxicity during cancer immunotherapy may be caused by other mechanisms or by other Abs that are not routinely measured [10]. In any case, thyroid autoimmunity in cancer patients treated with ICPIs is worth further investigation, in view of published findings suggesting a more favorable outcome in patients who develop autoimmune features during therapy with immunomodulatory agents (i.e., interferon or GVAX) [29, 30]. With regard to ICPIs, a few studies suggested a better outcome in those patients, in which a thyroid dysfunction occurred during treatment with anti PD-1 or anti PD-L1 molecules, in contrast to our findings [31–33]. However, a clear relationship with thyroid autoimmunity was not demonstrated. In addition, the heterogeneity of the series of patients in different studies (e.g., different types of cancer, different drugs) may likely affect the results of analyses addressing clinical outcomes. Admittedly, this is an interesting, yet not definitively solved, issue, so far.

Noteworthy, when all toxicities, which included symptoms suggesting a possible autoimmune response (e.g., fatigue, skin rash, itchiness, diarrhea, pneumonia, hypertransaminasemia/hyperbilirubinemia, hyporexia/anorexia) were considered, we observed a strong correlation between irAEs and a better outcome, in terms of both PFS and OS.

The fact that our study is retrospective did not allow a formal sample size calculation. Nevertheless, patients had been subjected to a strict follow-up program and the assessment of blood parameters during the scheduled hospital admissions for treatments allowed a standardized collection of data. Admittedly, the main strength of the study relies on the sample size, which represents to our knowledge the largest series of patients included in an original study, so far, outside of a clinical trial protocol, thus representing real-life data.

In summary, in this study we have investigated the presence of endocrine toxicities in a large series of cancer patients treated with the anti-PD1 nivolumab or pembrolizumab. The presence of a pre-existing endocrinopathy was a strong predictor of endocrine toxicity, which in most instances appeared in the first few months after the initiation of immunotherapy. Thyroid dysfunction was by far the most frequent endocrinopathy and both hypothyroidism, preceded or not by transient thyrotoxicosis, or thyrotoxicosis were observed. These alterations required specific pharmacological treatment in almost half of cases. Therefore, endocrine surveillance, and in particular thyroid function assessment, should be routinely performed in these patients.
